# Interproximal Reduction Facilitating Orthodontic Teeth Extraction

**DOI:** 10.7759/cureus.41403

**Published:** 2023-07-05

**Authors:** Uday Kumar Alle

**Affiliations:** 1 Orthodontics, Phoenix Hospital, Abu Dhabi, ARE

**Keywords:** malalignment, atraumatic extractions, safe exodontia, interproximal reduction, extraction of teeth, orthodontic extraction, dental surgeon, crowding of teeth

## Abstract

Extractions are routinely performed in orthodontics to gain space for teeth alignment. Crowded, malaligned, and overlapped teeth make it difficult for the dental surgeon to engage the beaks of the extraction forceps on the concerned tooth for extraction. An improper grip often leads to complications of instrument slippage, crown fracture, and more commonly, luxation of adjacent teeth. This article aims to help with atraumatic orthodontic extractions and reduce such complications. This case report examines an interproximal reduction technique using standard grit, taper, flat-end diamond bur (Mani TF-20, ISO 171/014, Mani, Inc., Tochigi, Japan) for the tooth to be extracted to create enough space for proper placement of the forceps and to prevent injury to adjacent structures. It can be useful for orthodontic extractions or other cases of tooth extractions with inadequate access.

## Introduction

Exodontia, or tooth extraction, is the painless removal of a whole tooth or tooth root from its socket with minimal trauma to the surrounding tissues so that the bone heals uneventfully, and no postoperative prosthetic complications arise [1]. Atraumatic exodontia is influenced to a large extent by the location and position of the tooth to be extracted. A properly aligned tooth has a conventional approach to extraction. However, crowded or other malaligned teeth may pose difficulty in the positioning of the forceps and the elevators. Complications of exodontia include injury to adjacent teeth, including fracture or dislodgement of adjacent tooth restoration, luxation of the adjacent tooth, and extraction of the wrong tooth. The dental surgeon typically employs various instruments or a surgical approach, such as flap surgery or transalveolar extractions, and sectioning of tooth or root [2] for adequate access to the operating site and to control the luxating forces. These are accompanied by various complications such as blood loss, poor healing, and soft and hard tissue loss [3]. There are several atraumatic extraction techniques introduced especially if the site is planned for a future dental implant.

Orthodontic treatment frequently includes tooth extraction to create space for the correction of crowded or proclined teeth. While avoiding trauma during the extraction procedure is the primary priority for both the patient and the dental surgeon performing it, the orthodontist also has a secondary goal, i.e., to prevent the cortical plates from fracturing during extraction, which could result in ridge narrowing [4]. Other complications of exodontia with poor access are instrument slippage, injury to the surrounding soft tissues, crown fracture, damaged cortical plates, soft tissue loss, and luxation of the adjacent tooth. Therefore, any method that facilitates tooth removal with minimal damage to the cortical plates is appreciated. A literature search done by the author revealed no special technique that addresses the extraction of a tooth from the location of crowded or misaligned teeth.

Interproximal reduction (IPR) is another prominent procedure that is routinely utilized in orthodontics to produce the space needed to align crowded teeth and improve occlusion. IPR involves the selective removal of enamel proximally to create space for tooth movement, a technique that was first introduced in the 1940s by Ballard for use on anterior teeth. It is the removal of enamel between teeth by mechanical means. A conservative IPR should not exceed 0.5 mm from any single interproximal surface [5]. This leaves sufficient enamel to resist decay and avoid sensitivity. Here, we adapted the IPR technique on the tooth to be extracted. IPR is performed on either side or both sides of a malposed tooth or until the contact point is broken, this gives the dental surgeon enough room for the engagement of the forceps and the elevator if necessary and prevents injury to adjacent structures. It also makes the procedure quick and atraumatic. However, the above guideline of IPR of 0.5 mm need not be followed if the tooth in question has to be extracted.

## Case presentation

**Figure 1:**
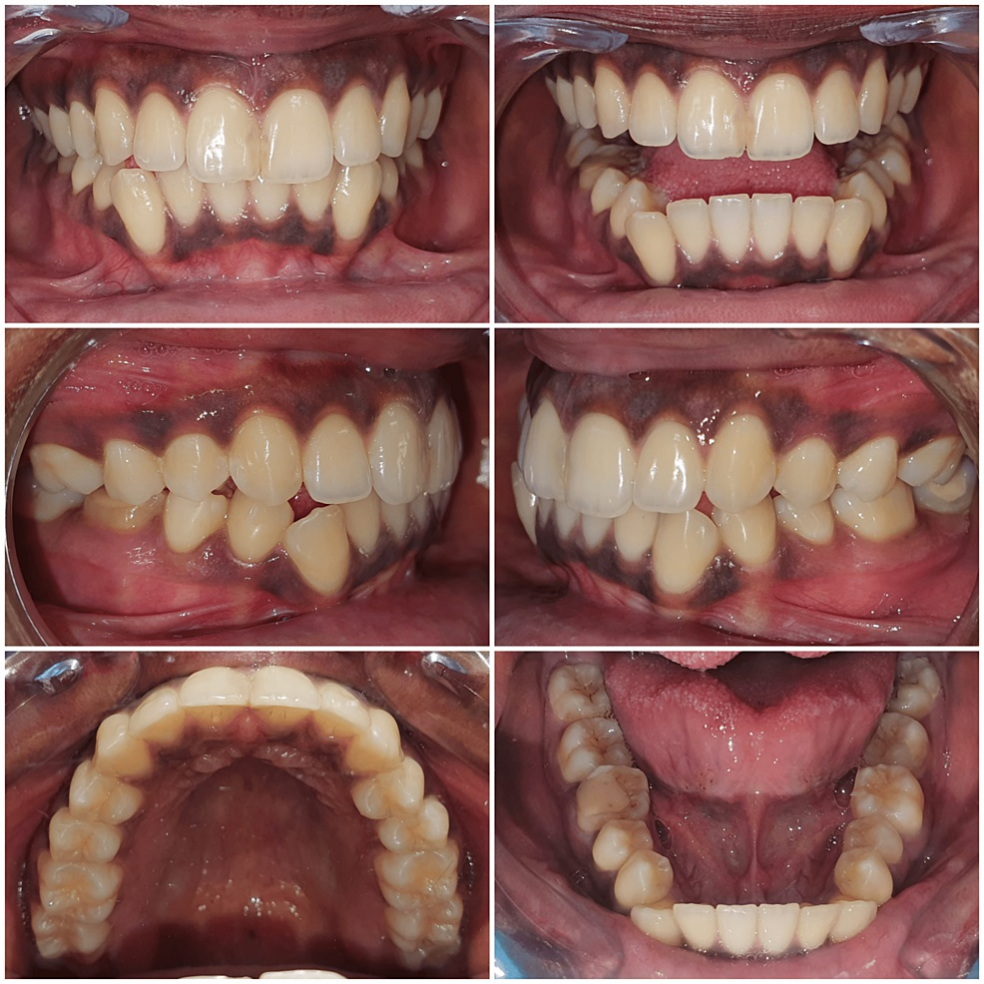
Pre-treatment intra-oral photographs of the patient

An 18-year-old female patient presented with a chief complaint of crooked lower front teeth. On clinical examination, the molars were in half unit class III relationship bilaterally, with a class I incisor and class III canine relation on class I skeletal base. The maxillary anterior teeth were proclined with severe crowding in the mandibular arch (Figure 1).

Radiographic investigation

**Figure 2:**
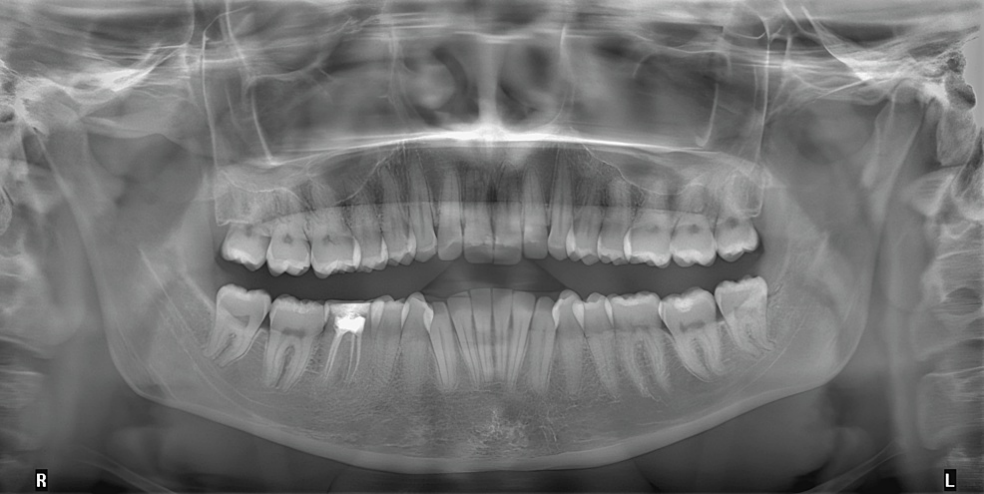
Pre-treatment orthopantomogram

Radiographic examination of an orthopantomogram (OPG) revealed complete adult dentition, previously root canal-treated lower right first molar tooth, and no other abnormalities were noticed (Figure 2).

Proposed treatment plan

The treatment plan included orthodontic tooth alignment of maxillary and mandibular teeth with the extraction of all the first premolars. The treatment focused on alleviating the mandibular teeth crowding, reducing the proclination of the maxillary anterior teeth, and achieving a class I molar and class I canine relationship.

Difficulties encountered while planning the extraction of lower first premolars

**Figure 3:**
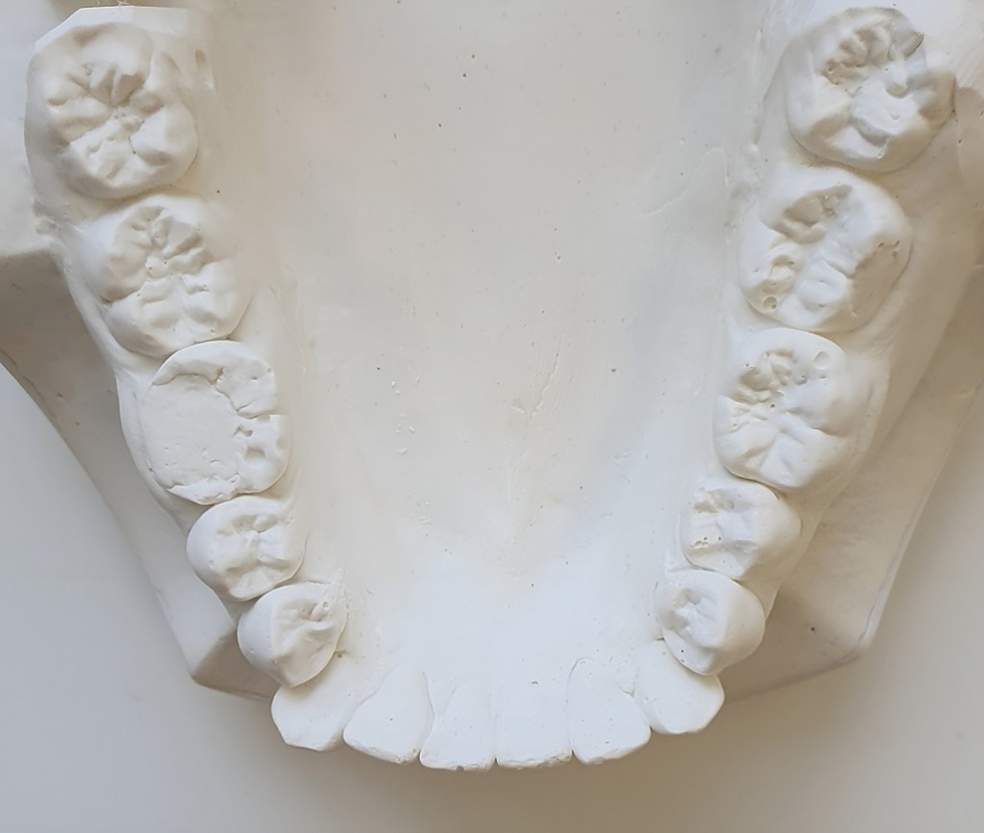
Lower arch study model (occlusal view)

The dental surgeon had difficulty engaging the beaks of the forceps used for lower first premolars extraction. The adjacent canine was labially positioned and distally tipped and the lower first premolars were lingually overlapping the canine, the lower arch study model occlusal view clearly shows the overlap of the lower first premolar lingually to lower canines (Figure 3). Although the forceps were able to engage in our case, the pressure from forcibly engaging the forceps could cause the subluxation of the canine. Here, we adopted the minimally invasive technique of mesial proximal reduction of the mandibular first premolars prior to extraction to establish adequate space for engaging forceps and the elevator and to avoid subluxation of adjacent teeth.

The extraction procedure

**Figure 4:**
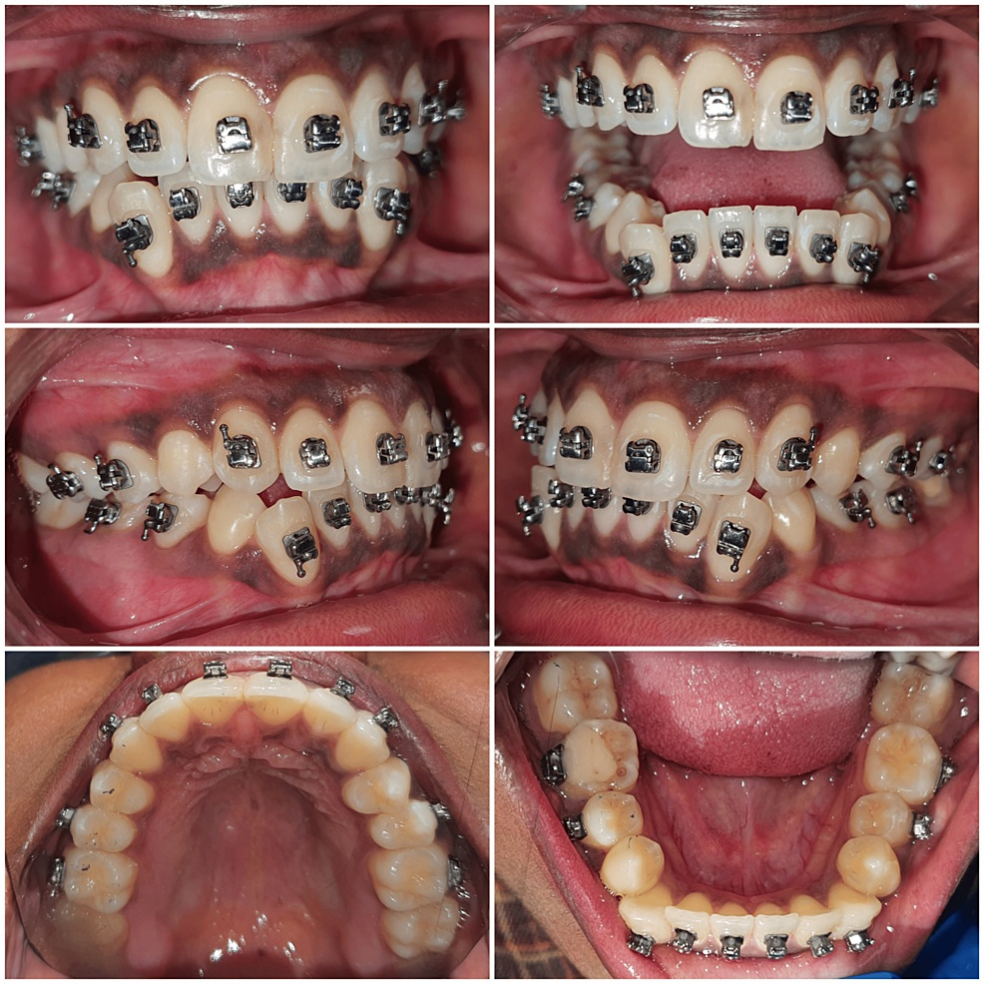
Brackets placed before extraction

Brackets were placed before extraction so that the archwire could be engaged immediately after extraction of the mandibular right and left premolars (Figure 4). Bonding procedures were performed prior to orthodontic tooth extraction so that it reduces the waiting period of 15 days if the extraction was done prior to bonding.

The technique

Here, we adapted the technique of mesial proximal reduction of the first premolar prior to extraction to create adequate space for the tooth forceps and the elevator and to prevent subluxation of adjacent teeth.

**Figure 5:**
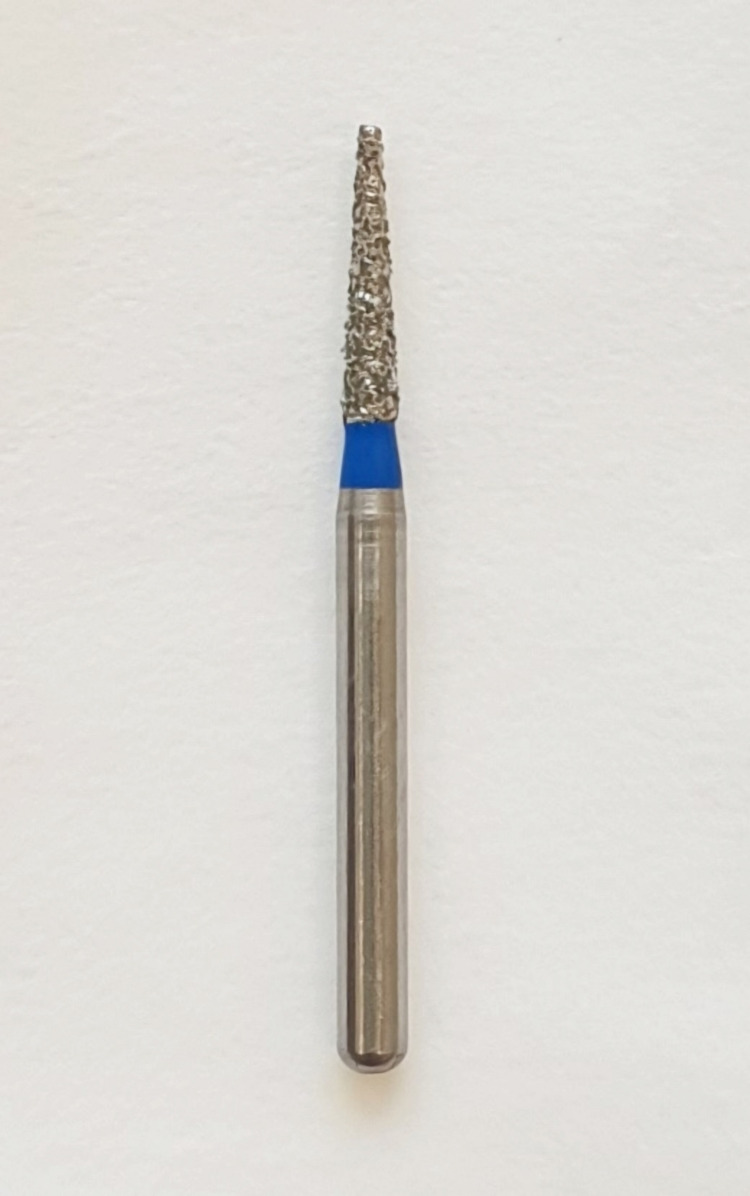
Standard grit, taper, flat-end diamond bur (Mani TF-20, ISO 171/014)

The armamentarium used included the following: (1) a high-speed handpiece (NSK Dental Air Rotor Handpiece Standard, NSK Dental, Tochigi, Japan); (2) standard grit, taper, flat-end diamond bur (Mani TF-20, ISO 171/014, Mani, Inc., Tochigi, Japan) (Figure 5); (3) local anesthetic spray (Nummit Spray, ICPA Health Products Ltd., Mumbai, India); (4) injectable local anesthesia (1:80,000 epinephrine) (Lignox 2% with adrenaline 1:80000 30 ml); (5) lower premolar extraction forceps (Hu-Friedy, Chicago, IL); (6) periosteal elevator (Hu-Friedy); (7) saline irrigation; (8) gauze.

**Figure 6:**
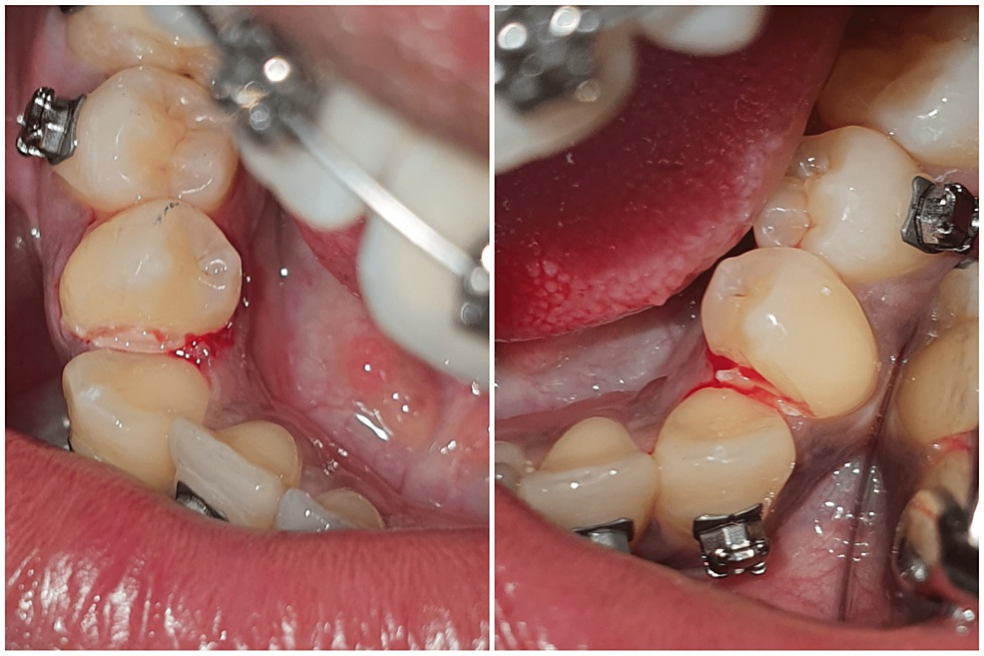
Interproximal reduction of the right and left lower first premolars

An inferior alveolar and lingual nerve block was administered with respect to the patient's mandibular right side. After reviewing the subjective symptoms, a metal matrix band (Tofflemire Matrix Bands, Patterson®, Saint Paul, MN) was placed on the distal surface of the canine to prevent any iatrogenic damage to the tooth. A standard grit, taper, flat-end diamond bur (Mani TF-20, ISO 171/014) was utilized on the mesial side of the first premolar to break the contact between the canine and the premolar; the space was created, and the premolar overlapping was removed (Figure 6).

**Figure 7:**
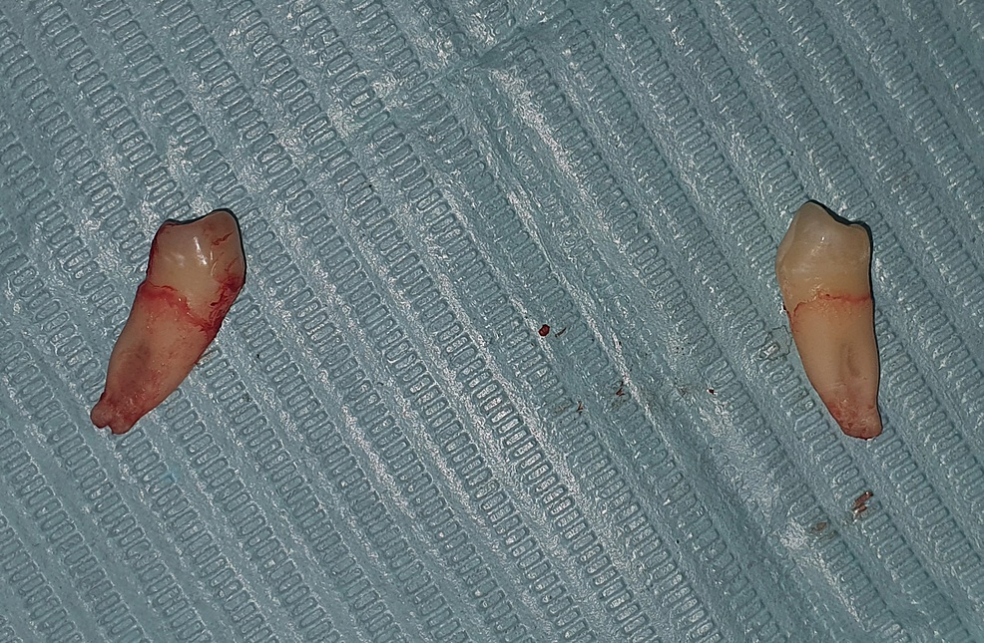
Extracted right and left mandibular first premolars

**Figure 8:**
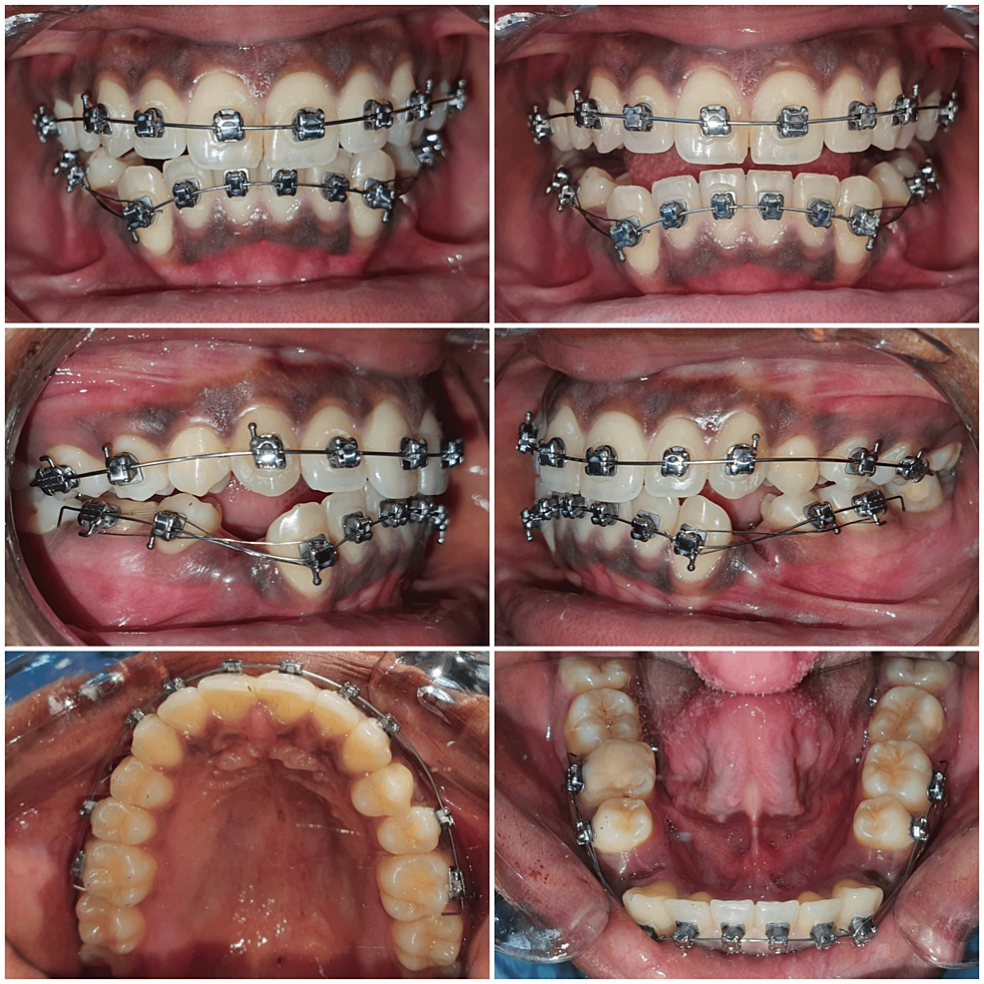
Post-extraction follow up

The periosteal elevator (Hu-Friedy) was utilized to break the periodontal ligament (PDL) fibers and for the elevation of soft tissues, and the lower premolar extraction forceps (Hu-Friedy) were engaged as apically as possible, without any difficulty. The tooth was successfully extracted without causing any injury to the adjacent canine. The extraction site was cleaned and disinfected. The archwire was engaged in the brackets. A gauze pack was placed at the extraction site. The patient was prescribed routine non-steroidal anti-inflammatory drugs (NSAIDs) for the alleviation of post-extraction pain. The same procedure was followed for the extraction of the left mandibular first premolar shortly after the completion of the extraction on the right side (Figure 7). The patient was recalled after a week (Figure 8) and the extraction sockets were healed uneventfully.

## Discussion

Professional disagreement and debate over the issue of extracting permanent teeth as part of corrective orthodontics have persisted for years. However, it is integral to the treatment of dental crowding with orthodontics. Furthermore, orthodontists may recommend extracting a tooth if there is an imbalance in the jaw's growth. Tooth extraction is commonly a treatment consideration in the orthodontic management of dental crowding. In addition, orthodontists may consider extraction in cases of jaw growth discrepancy, such as Angle class II relationships, and in various other conditions, such as tooth pathology or injury to the tooth [6]. Orthodontic extractions have to be atraumatic to preserve the alveolar bone, the soft tissues, and the health of the adjacent teeth. Physics forceps are an effective method of atraumatic extraction of premolars as they reduce the intraoperative time significantly and have comparable clinical outcomes as the conventional forceps and are associated with few complications [7]. Severe crowding and tipped overlapping teeth make this more difficult. The use of lighter beaked forceps allows the forceps beaks to be placed on the tooth surface but does not prevent adjacent overlapped teeth from subluxation.

Choosing the correct technique and following fundamental principles lead to an atraumatic extraction. The three fundamental requirements for a good extraction are (a) adequate access and visualization of the surgical field; (b) an unimpeded pathway for the removal of the tooth; and (c) use of controlled force to luxate and remove the tooth [2].

During the extraction of a tooth, five different sequential motions were performed using forceps. These are apical pressure, buccal/labial pressure, palatal/lingual pressure, rotational pressure, and tractional forces. Tooth extraction from crowded teeth makes all these five motions difficult to perform and there is a risk of subluxation of adjacent teeth.

There are several atraumatic extraction techniques introduced, especially if the site is planned for a future dental implant, such as reducing contact areas, atraumatic extraction by using an atraumatic extraction kit [8], socket shield technique, and the Benex extraction system. A method to reduce the damage is to limit the connecting area of the tooth that needs to be extracted [9].

Several studies aim to bypass surgical extractions in orthodontics to prevent bone and soft tissue loss and post-surgical complications. A case study discussed the use of a removable appliance to separate the inferior alveolar nerve from the proximity of an impacted third molar [10]. Thinking along similar lines, this study showed how a minor procedure like IPR, which is routinely done in conventional orthodontics and aligner therapy, can be used in the case of a severely malposed tooth to create adequate space for the engagement of the extraction forceps, avoiding unnecessary surgical involvement [11]. Vertical extraction systems may be used with a high success rate for the extraction of teeth unsuitable for forceps extraction [3].

This study demonstrated how a minor procedure like interproximal reduction (IPR), which is routinely utilized in conventional orthodontics and aligner therapy for teeth alignment, can be employed in a different scenario like difficult exodontia. A surgical extraction can be avoided by creating adequate space on one or both sides of the tooth to be extracted. The method prevents inadvertent forces on the adjacent tooth, which can result in subluxation, injury to the interdental papilla, and dislodgement of a crown or restoration. In addition, the method is patient-friendly and painless, with quick recovery. The technique does not necessitate an elaborate armamentarium and can be performed quickly with a regularly available dental clinic setup. It can be utilized for orthodontic as well as conventional extractions from sites of crowded, malaligned, and overlapped teeth.

The IPR technique is utilized widely and safely in orthodontics for tooth alignment. The IPR technique on the tooth supposed to be extracted makes it safer and can be easily adapted in any such clinical scenario without side effects with minimal available armamentarium in daily clinical practice.

## Conclusions

The orthodontic tooth extraction from the crowded jaw was successfully performed utilizing the interproximal reduction technique on the tooth supposed to be extracted with minimal complications. The technique of employing IPR of the proposed tooth for extraction for orthodontic treatment is a quick and practical alternative for safe exodontia. It facilitates the extraction of a tooth from a crowded or malaligned segment of the jaw easily. It also safeguards the health of the adjacent structures.

The procedure is designed to avoid complications such as adjacent tooth subluxation and tissue damage. The study demonstrates that this minimally invasive approach can be effective, patient-friendly, and results in a quick recovery. It suggests that interproximal reduction, commonly used for creating space in orthodontics, can also be beneficial in challenging extractions of crowded and malaligned teeth.

## References

[1] Howe GL (1962). Some complications of tooth extraction: lectures delivered at the Royal College of Surgeons of England on 27th April 1961. Ann R Coll Surg Engl.

[2] Jain A Principles and techniques of exodontia. Oral and Maxillofacial Surgery for the Clinician.

[3] Hong B, Bulsara Y, Gorecki P, Dietrich T (2018). Minimally invasive vertical versus conventional tooth extraction: an interrupted time series study. J Am Dent Assoc.

[4] Rai AK, Yadav B (2016). Facilitating orthodontic teeth extraction-a technique suggestion. Saudi J Dent Res.

[5] Blank JT (2010). Revolutionizing interproximal enamel reduction. Inside Dent.

[6] Peck S, Peck H (1979). Frequency of tooth extraction in orthodontic treatment. Am J Orthod.

[7] Kapila S, Kaur T, Bhullar RS, Sandhu A, Dhawan A, Kaur A (2020). Use of physics forceps in atraumatic orthodontic extractions of bilateral premolars: a randomized control clinical study. J Maxillofac Oral Surg.

[8] Singla Y, Sharma R (2020). Latest trends in atraumatic extraction of teeth. Int J Appl Dent Sci.

[9] Saha A, Jalaluddin M, Kar S, Sarang M (2022). A review on atraumatic extraction for implant placement. Int J Sci Res.

[10] Wang Z, Liu Z, Shi Y, Fang D, Li S, Zhang D (2019). A novel orthodontic extraction method for removal of impacted mandibular third molars in close proximity to inferior alveolar nerve. J Oral Maxillofac Surg.

[11] Pindoria J, Fleming PS, Sharma PK (2016). Inter-proximal enamel reduction in contemporary orthodontics. Br Dent J.

